# Regulatory role of VvsB protein on serine protease activity of VvsA in *Vibrio vulnificus*

**DOI:** 10.1093/femsle/fnae053

**Published:** 2024-07-17

**Authors:** Tomoka Kawase, Anusuya Debnath, Keinosuke Okamoto

**Affiliations:** Graduate School of Medicine, Dentistry and Pharmaceutical Sciences, Okayama University, Tsushima-Naka, Kita-Ku, Okayama 700–8530, Japan; Department of Biotechnology, Brainware University, Kolkata Barasat, West Bengal 700125, India; Graduate School of Medicine, Dentistry and Pharmaceutical Sciences, Okayama University, Tsushima-Naka, Kita-Ku, Okayama 700–8530, Japan

**Keywords:** RTS system, *in vitro* cell-free translation system, PU, Proteinase unit, VvsA, *Vibrio vulnificus* serine protease, SD, Shine-Dalgarno sequence

## Abstract

**Background:** *Vibrio vulnificus* NCIMB2137, a Gram-negative, metalloprotease negative estuarine strain was isolated from a diseased eel. A 45 kDa chymotrypsin-like alkaline serine protease known as VvsA has been recently reported as one of the major virulence factor responsible for the pathogenesis of this strain. The *vvsA* gene along with a downstream gene *vvsB*, whose function is still unknown constitute an operon designated as *vvsAB*. **Objective:** This study examines the contribution of VvsB to the functionality of VvsA. **Method:** In this study, VvsB was individually expressed using Rapid Translation System (RTS system), followed by an analysis of its role in regulating the serine protease activity of VvsA. **Result:** The proteolytic activity of VvsA increased upon the addition of purified VvsB to the culture supernatant of *V. vulnificus*. However, the attempts of protein expression using an *E. coli* system revealed a noteworthy observation that protein expression from the *vvsA* gene exhibited higher protease activity compared to that from the *vvsAB* gene within the cytoplasmic fraction. These findings suggest an intricate interplay between VvsB and VvsA, where VvsB potentially interacts with VvsA inside the bacterium and suppress the proteolytic activity. While outside the bacterial milieu, VvsB appears to stimulate the activation of inactive VvsA. **Conclusion:** The findings suggest that *Vibrio vulnificus* regulates VvsA activity through the action of VvsB, both intracellularly and extracellularly, to ensure its survival.

## Teaser

When VvsA transitions into its active state, the presence of VvsB and its binding activity suppresses the activation of VvsA within the extracellular milieu. The protein expression levels of VvsA and VvsB appears to be stringently regulated, contributing to the environmental adaptability of *Vibrio vulnificus* NCIMB 2137.

## Introduction

Proteases produced by bacteria exhibit toxicity by degrading host proteins at the site of infection and by disrupting the host signaling system (Miyoshi and Shinoda 2000, Shinoda and Miyoshi 2011). However, these proteases also pose a threat to bacterial cells themselves, as they degrade cellular constituents. Thus, it becomes crucial for bacteria not only to regulate the production rate of proteases but also to express their activity at specific sites. Therefore, it is imperative to devise methods that prevent the produced proteases from decomposing cellular components. However, protease expression levels are typically low in bacteria, and the intricate regulatory mechanisms governing the expression of active bacterial proteases remain incompletely understood.

*Vibrio vulnificus* is a prevalent estuarine bacterium characterized by its Gram-negative and halophilic nature. Despite its common occurrence, this bacterium transforms into an opportunistic human pathogen, demonstrating high lethality and being implicated in seafood-related fatalities across various countries (Jones and Oliver 2009). The resultant primary septicemia from *V. vulnificus* infection is particularly deadly, with an average mortality rate surpassing 50%.

The potential virulence factor termed as VvsA, a serine protease, has been reported in *V. vulnificus* strains isolated from a diseased eel (Tison et al. 1982, Senoh et al. 2005, Wang et al. 2008). VvsA is demonstrated to be a chymotrypsin-like protease. It has been shown that 45 kDa mature VvsA is derived from the 59 kDa intermediate product after removal of the C-terminal 14 kDa polypeptide. This VvsA is an orthologue of an extracellular protease produced by *Vibrio parahaemolyticus*, a causative agent responsible for both human wound infection as well as gastroenteritis (Chakraborty et al. 1997). Hence, it is reasonable to infer that VvsA may play a role in the distinctive skin damage observed in human infections. Additionally, it could serve as a virulence factor in eel vibriosis, characterized by both external and internal hemorrhages (Lee et al. 2002). VvsA is encoded in an operon which contain two genes *vvsA* downstream gene *vvsB*. The *vvsA* and *vvsB* were separated by only 6 bp. The homologue of VvsB precursor (115aa) was found in several *Vibrio* species of the serine protease family. This identity was low (36.5–56.5%). The 9.1 kDa VvsB was derived by removing of the C-terminal 2.9 kDa signal peptide. But its function is not yet known (Miyoshi et al. 2012).

In recent times, cell-free protein synthesis systems have emerged as a powerful technology platform for the rapid, efficient, and cost-effective analyses of actions of proteins (Schwarz et al. 2008). Sometimes it is being difficult to express some bacterial proteins in living cells because of their potent toxicity. So, the *in vitro* protein synthesis machinery is a technical boon to overcome such limitations.

This study aims to investigate the role of VvsB in regulating the functionality and activity of VvsA, a 45 kDa chymotrypsin-like alkaline serine protease, in *Vibrio vulnificus* NCIMB2137. Specifically, the research seeks to understand how VvsB influences the proteolytic activity of VvsA both within the bacterial cell and in the extracellular environment, thereby contributing to the pathogen's virulence and survival.

## Materials and methods

### Bacterial strains and growth conditions

*Vibrio vulnificus* NCIMB2137 was isolated from eel. The bacteria were grown in Luria-Bertani medium (1.0% Bacto tryptone, 0.5% Bacto yeast extract, 1.0% NaCl) at 37°C with appropriate antibiotics. The genomic DNA of *V. vulnificus* was extracted as described earlier (Senoh et al. 2005).

### The DNA template for cloning of *vvsB* gene encoding serine proteases for RTS system

The rapid translation system (RTS100 *E. coli* HY Kit, 5PRIME Inc., Gaithersburg, MD, USA) was used as a cell-free translation system for the expression of proteins *in vitro*. In this study, a linear DNA fragment (*vvsB*) inserted into the RTS expression vector pIVEX2.4d (RTS^TM^ pIVEX His-tag 2^nd^ Generation Vector Set Short Instruction 5PRIME Inc., Gaithersburg, MD, USA) to generate a hybrid plasmid. Genomic DNA of *Vibrio vulnificus* NCIMB2137 strain was used as a PCR template for the amplification of the *vvsB* gene (GenBank accession number, AB509375), using primers listed in Table 1.

**Table 1.** Primers used in this study.

Primer	Sequence (5'→3')
vvsB-S	GC**GCGGCCGC**AATAACAAGGGGTTC
vvsB-A	CG**GACGTC**TTACTGGGTGTACAGTTGCCTAACA
pBlue-sacI-*vvsA*	GGGCGAATTG**GAGCT**ATGCATTACACAACAACAAAAGC
pBlue-kpnI-*vvsA*-R	ACAAAAGCTG**GGTAC**TTACTGGAAGGTTAATGTCCAG
pBlue-kpnI-*vvsB*-R	ACAAAAGCTG**GGTAC**TTACTGGGTGTACAGTTGCC
Prom-*vvsA*-sacI	GGGCGAATTG**GAGCTC**TTCATAGAACTATCTTCTTATGTTTTG

Underlined: Overlapping region

Bold: The restriction enyme site on the vector multiple-cloning site (MCS)

Next, PCR amplified *vvsB* (282 bp) gene without a signal peptide sequence was cloned in the expression vector pIVEX2.4d (Kawase et al. 2022). It was then transformed into *E. coli* DH5α strain and the positive transformants were selected on ampicillin supplemented LB plates. The recombinant plasmid was purified using Quantum Prep Plasmid mini Prep (Bio-Rad Laboratories, Hercules, CA, USA).

### In vitro synthesis of proteins by RTS

The synthesis of serine protease was done using circular DNA (0.5 µg) expressed in *in vitro* system. The 50μl reaction solution was set up according to manufacturer's protocol (RTS100 *E. coli* HY kit, 5PRIME, USA). The coupled transcription-translation reaction was carried in a RTS ProteoMaster (Roche) at 25ºC, 400 rpm for 6 h. The protease inhibitor was added only if metalloprotease was expressed. The serine protease inhibitor used was the cOmplete, Mini (Roche & Sigma Aldrich Japan).

### Purification of His-tagged protein

For purification of the RTS product, Capturem His-tagged Purification Mini prep Kit (TaKaRa, Japan) was used. 200 µl of the reaction mixture was purified according to the manufacture's protocol.

### Assay for protease

Azocasein at a concentration of 5 mg/ml served as the substrate for assessing protease activity. The protease activities of RTS products were evaluated using the procedure outlined by Miyoshi et al. (1987). Absorbance readings were taken at 440 nm, with one protease unit (PU) defined as the quantity of enzyme capable of hydrolyzing 1 µg of azocasein within 1minute. Protein amounts was measured by using a spectrophotometer.

### Preparation of recombinant proteins and their extraction

The *vvsA* or *vvsAB* genes (accession number AB509375)with or without operator/promoter (O/P) and Shine-Dalgarno sequence (SD) derived from *V. vulnificus* NCIMB2137, were amplified by PCR. The primer sets Prom-*vvsA*-sacI/pBlue-kpnI-*vvsA*-R (2,123 bp) or pBlue-sacI-*vvsA*/pBlue-kpnI-*vvsA*-R (1,692 bp) for *vvsA*, and pBlue-sacI-*vvsA*/pBlue-kpnI-*vvsB*-R (2,409 bp) or Prom-*vvsA*-sacI/pBlue-kpnI-*vvsB*-R (2,476 bp) for *vvsAB* were used (Table 1). The PCR products were ligated into the pBluescript IIKS + vector, which includes the Amp^r^ marker, employing the HiFi DNA Assembly Master Mix kit (NEW ENGLAND Bio Labs). Subsequently, *E. coli* HB101 (TaKaRa Bio, Otsu, Japan) was subjected to transformation with these recombinant plasmids and the positive transformants were selected on ampicillin supplemented LB plates. The protein fractions were obtained using the EzBactYeast Crusher kits (ATTO, Tokyo, Japan).

### SDS-PAGE

Each RTS products or His-tag purified protein solution (15 µl) were suspended in SDS sample buffer (10% SDS, 70% glycerol, 0.05% bromophenol blue, 0.5 M Tris-HCl, 5% 2-mercaptoethanol) and the sample was heated at 100ºC for 5 min. 20 µl of this sample was then subjected to electrophoresis. After SDS-PAGE, the proteins were stained with Coomassie brilliant blue R-250.

### N-terminal amino acid sequence

The proteins separated by SDS-PAGE were transferred to a PVDF membrane (BIO-RAD, USA) and stained with Amido Black T. N-terminal amino acid sequence was determined using an Applied Biosystem Precise Sequencer (Applied Biosystem, Foster City, CA, U.S.A.)

## Results

### Purification of VvsB synthesized by RTS system

**Figure 1.**
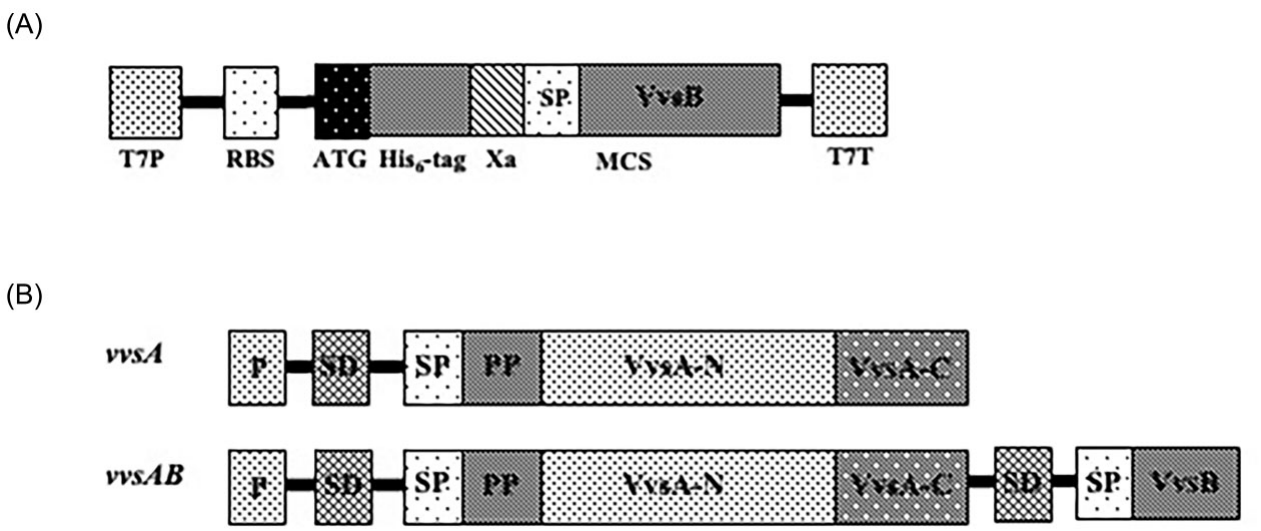
Functional elements of cloning map. It shown the *vvsA* and *vvsAB* gene of *V. vulnificus*. A: Linear *vvsB* inserted into the pIVEX2.4d expression vector. T7P; T7 Promoter, RBS; Ribosomal binding site, ATG; Start codon, His_6_-tag; gene for the tag of His_6_, Xa; Factor Xa restriction protease cleavage site, SP; Signal peptide, MCS; Multiple cloning site for the insertion of the target gene, T7T; T7 Terminator. B: Arrangement of the functional proteins of *vvsAB* fragments inserted into the vector. P; Promoter, SD; Shine-Dalgano sequence, SP; Signal peptide, PP; Propeptide, VvsA-N; N-terminal VvsA, VvsA-C; C-terminal VvsA.

**Figure 2.**
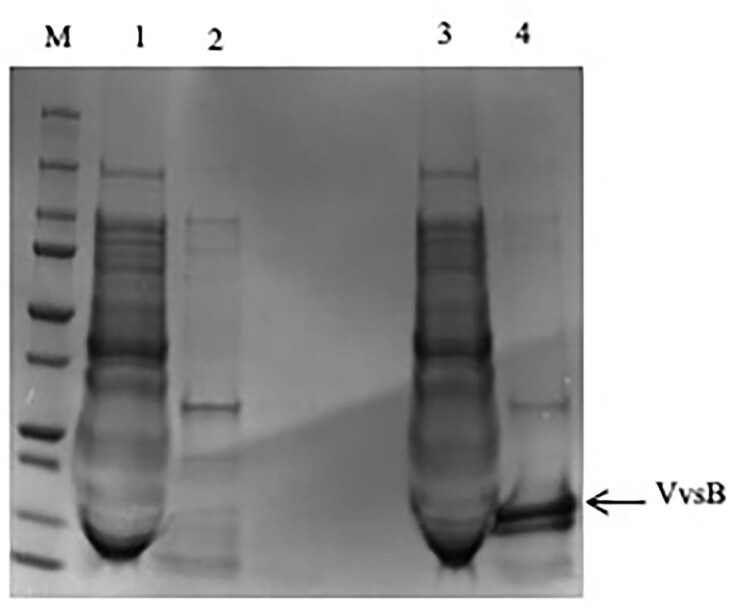
SDS-PAGE followed by Coomassie brilliant blue staining of the gel. The protein produced by RTS system at 25°C, 6hr using the pIVEX2.4d vector and the hybrid (*vvsB* inserted to pIVEX2.4d vector) plasmids. The RTS products were subjected to SDS-PAGE. Lane M, Protein molecular weight marker (250, 150, 100, 75, 50, 37, 25, 20, 15, and 10 kDa); lane 1, pIVEX2.4d vector; lane 2, His-tag purification of pIVEX2.4d vector expressed protein; lane 3, circular *vvsB* (11.7 kDa, SGSHHHHHHSSGIEG); lane 4, His-tag purification of circular *vvsB* expressed protein.

The protein synthesis was performed using circular *vvsB* DNA (Fig. 1A) in RTS system at 25°C for 6 h. The RTS reaction mixture was applied to His-tag column to get purified VvsB. In Fig. 2, lane 3 showed VvsB purification done by His-tag column after synthesized by RTS. The N-terminal amino acid sequencing identified the sequence SGSHHHHHHSSGIEG, indicating the presence of the histidine-tagged VvsB. Thus, the expression of VvsB was confirmed.

### The role of VvsB in *in vivo* system during the synthesis of serine protease VvsA and later on its proteolytic activity

We employed the traditional approach of protein expression within the bacterial system to investigate the function of VvsB. In Fig. 1B, a schematic of the genes inserted into the pBluescript IIKS + vector was presented. The constructs were generated with and without O/P and SD sequence derived from *V. vulnificus* NCIMB2137 (Fig. 1B).

**Figure 3.**
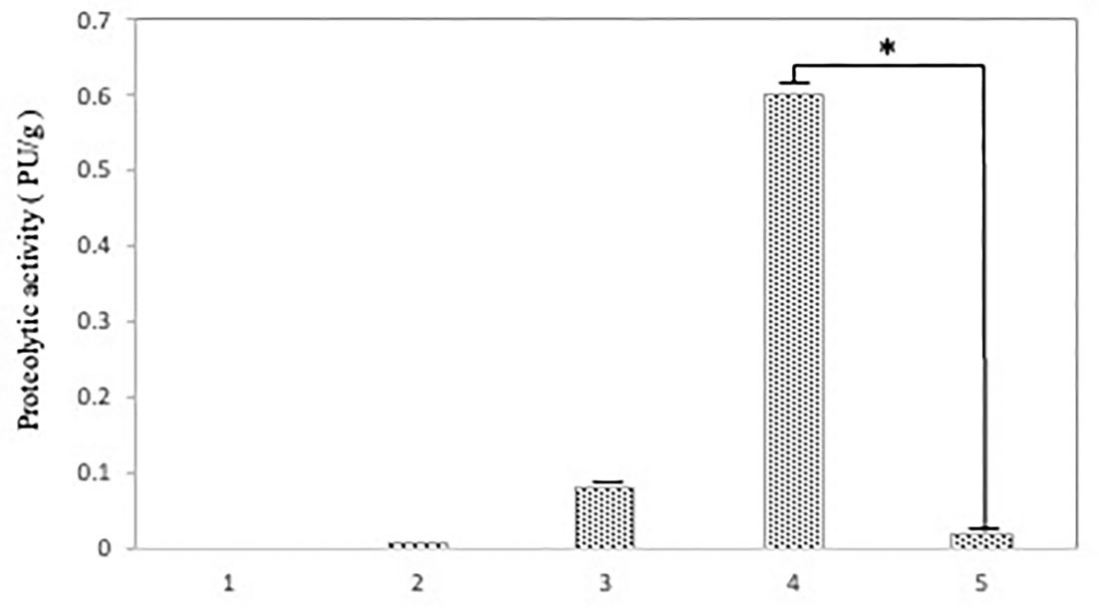
The proteolytic activity of proteins produced from *E. coli* recombinants. Azocasein (5 mg/ml) was used as a substrate for the measurement of protease activity per protein amounts. The purified protein solution after the reaction then mixed with an equal volume of 0.5 M NaOH and the absorbance was measured at 440 nm. One protease unit (PU) was defined as the amount of enzyme hydrolyzing 1 µg of azocasein in 1 min. Lane 1, pBluescript IIKS + vector; lane 2, VvsA produced by *vvsA* into the pBluescript IIKS + vector; lane 3, Both VvsA and VvsB produced by *vvsAB* into the pBluescript IIKS + vector; lane 4, VvsA produced by *vvsA* including O/P and SD derived from *V. vulnificus* NCIMB2137 inserted into the pBluescript IIKS + vector; lane 5, Both VvsA and VvsB produced by *vvsAB* including O/P and SD derived from *V. vulnificus* NCIMB2137 inserted into the pBluescript IIKS + vector. The data is the mean + S.D. of three or more experiments. The asterisk (*) indicates the significant difference (*P* < 0.05).

Subsequently, protein fractions were extracted from these recombinants, followed by the measurement of proteolytic activity. While the periplasmic protein fraction exhibited no activity (data not shown), the cytoplasmic fraction displayed proteolytic activity (Fig. 3). Notably, the protein product from the *vvsA* gene exhibited higher activity compared to the protease derived from the *vvsAB* gene (Fig. 3, lanes 3 and 4).

We found that the recombinants including O/P and SD derive from *V. vulnificus* NCIMB2137 (Fig. 3, lane 3 and 4) showed higher activity as compared to the constructs without these elements (Fig. 3, lane 1 and 2). This result suggests that *V. vulnificus* NCIMB2137 specific O/P and SD sequence was essential for the production and activation of VvsA. It also indicates that the way VvsB might controls VvsA production is specific for *V. vulnificus* that could not be possible to mimic in other bacterial system such as *E. coli* (Kawase et al. 2022).

**Figure 4.**
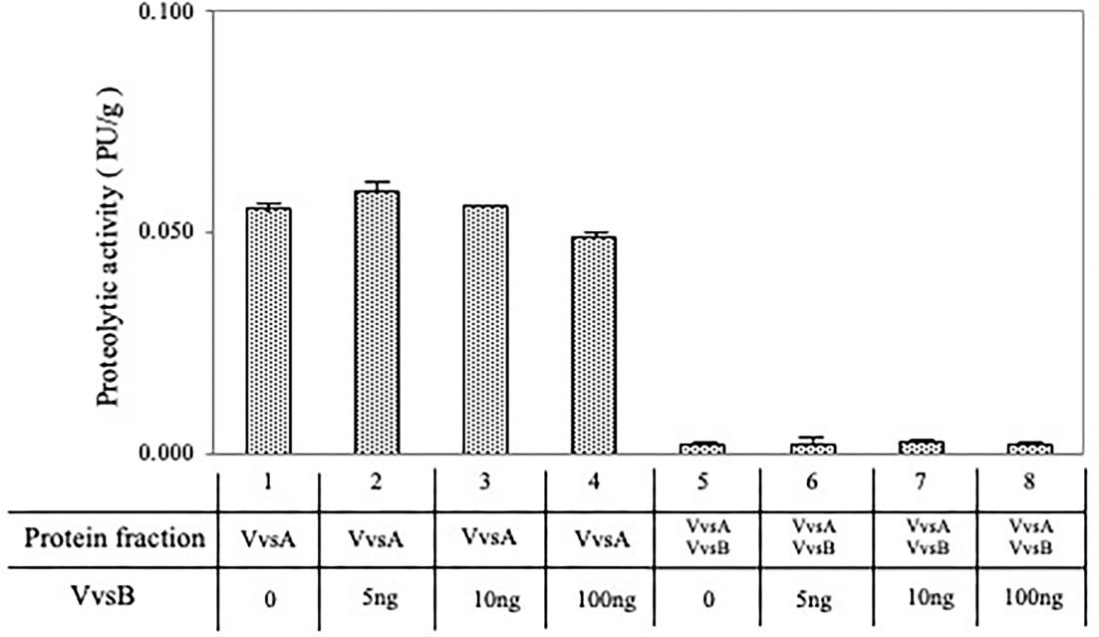
The effect of VvsB on the proteolytic activity of proteins produced from *E. coli* recombinants. VvsA produced by *vvsA* including O/P and SD derived from *V. vulnificus* NCIMB2137 inserted into the pBluescript IIKS + vector. Both VvsA and VvsB produced by *vvsAB* including O/P and SD derived from *V. vulnificus* NCIMB2137 inserted into the pBluescript IIKS + vector. Azocasein (5 mg/ml) was used as a substrate for the measurement of protease activity per protein amounts. The purified protein solution after the reaction was mixed with an equal volume of 0.5 M NaOH and the absorbance was measured at 440 nm. One protease unit (PU) was defined as the amount of enzyme hydrolyzing 1 µg of azocasein in 1 min. Lane 1, VvsA; lane 2, VvsA added 5 ng of the purified VvsB; lane 3, VvsA added 10 ng of the purified VvsB; lane 4, VvsA added 100 ng of the purified VvsB, lane 5, VvsA and VvsB; lane 6, VvsA and VvsB added 5 ng of the purified VvsB; lane 7, VvsA and VvsB added 10 ng of the purified VvsB; lane 8, VvsA and VvsB added 100 ng of the purified VvsB. The data is the mean + S.D. of three or more experiments.

Next, we tried to measure the protease activity of protein produced from *E. coli* recombinants, in the presence of purified 5 ng or 100 ng VvsB (Fig. 4). *E. coli* recombinants containing O/P and SD sequences derived from *V. vulnificus* NCIMB2137, which had higher protease activity, were used. The results showed that the recombinant VvsA had higher activity than the recombinant VvsAB (*t*-test for comparison at each concentration shows significant difference at *P* < 0.05). The serine protease activity was observed to decrease gradually with the addition of purified VvsB to the *vvsA* recombinant. However, this reduction was not very much significant. The results also showed a slight decrease in proteolytic activity with the addition of ≥ 10-100 ng of purified VvsB. In contrast, co-expression of VvsA and VvsB resulted in a significant reduction in serine protease activity. This observation suggests that VvsB acts as a weak inhibitor of serine protease activity.

### Effect of VvsB on proteolytic activity of VvsA in *in vitro*

**Figure 5.**
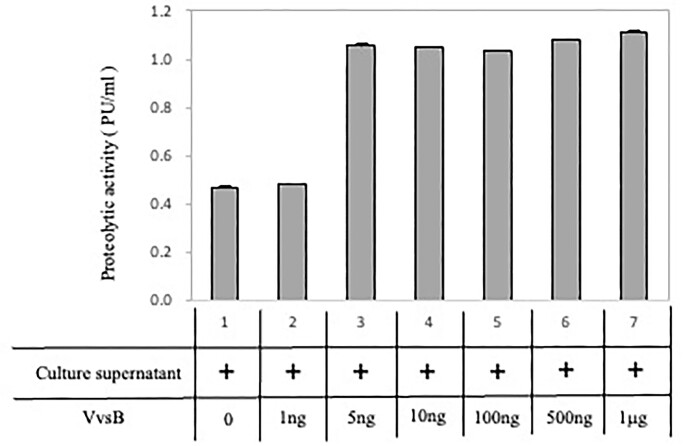
The effect of the proteolytic activity in culture supernatant added VvsB. Azocasein (5 mg/ml) was used as a substrate for the measurement of protease activity. One protease unit (PU) was defined as the amount of enzyme hydrolyzing 1 µg of azocasein in 1 min. The culture supernatant was collected at early stationary phase at 30°C. The *Vibrio vulnificus* culture supernatant and each VvsB (RTS product 0.67 µg/µl) mixture (100 µl) was measured at 30°C for 3 h. Lane 1, no add; lane 2, VvsB 1 ng; lane 3, VvsB 5 ng; lane 4, VvsB 10 ng; lane 5, VvsB 100 ng; lane 6, VvsB 500 ng; lane 7, VvsB 1μg. The data is the mean + S.D. of three or more experiments.

Next, we investigated the impact of purified VvsB on the proteolytic activity of VvsA-containing culture supernatant from *V. vulnificus* NCIMB2137. As illustrated in Fig. 5, the proteolytic activity of the supernatant was assessed in the presence of 1 ng to 1μg of purified VvsB. Surprisingly, proteolytic activity was maximal at 5 ng of VvsB, suggesting that the addition of VvsB did not act as an extracellular inhibitor (Fig. 5). Therefore, it is suggested that VvsB inhibits VvsA activity intracellularly to prevent autolysis, while it may facilitate VvsA activation outside the cell.

## Discussion

The *vvsA* gene together with *vvsB* gene constitutes an operon, where Shine-Dalgarno sequence of *vvsB* gene overlaps with the stop codon (TTA) of *vvsA* gene. Earlier we had reported that even though VvsB can be synthesized by RTS system, active VvsA cannot be synthesized (Kawase et al. 2022).

In general, a chaperone is a protein that aids in the proper folding of an immature protein to become functionally active. Once the correct folding is achieved, the chaperone protein dissociates from the mature protein (Eder et al. 1993, Cunningham and Agard 2003).

Since VvsA and VvsB have signal peptides, it is likely that these two proteins associate in the periplasm. However, the direct interaction between VvsA and VvsB in periplasm has not been analyzed yet. In contrast to general notion, we speculate that the interaction between VvsB and mature VvsA results into suppression in the activity of VvsA. In support of this speculation, it was found that VvsB can bind to the active form of VvsA and suppress its activity (Fig. 3). This suggests that VvsB acts as a weak inhibitor, but it is necessary to reseach moreover.

In Fig. 3, the *vvsA* and *vvsAB* genes were recombined into the protein expression vector pBluescript IIKS+, and the effect on serine protease activity was examined. We also conducted a comparative experiment with and without O/P and SD sequences derived from *V. vulnificus* NCIMB2137. These results showed that the serine protease activity was increased in the *vvsA* recombinant compared to the *vvsAB* recombinant (Fig. 3, lanes 3 and 4). Furthermore, the activity was increased in the strain containing genes containing O/P and SD sequences derived from *V. vulnificus* NCIMB2137 (Fig. 3, lanes 3 and 4). This also revealed that VvsB regulates VvsA production, and that the *Vibrio*-derived O/P and SD sequences are required to increase VvsA activity.

VvsA is a bacterial serine protease believed to primarily degrade host-derived proteins surrounding the bacteria into an ingestible form for nutrient acquisition (Lee et al. 1999). For VvsA to exhibit its enzymatic activity, it must be expressed at the appropriate site. However, the survival of the bacterium might get threatened if serine proteases accumulate excessively in inappropriate locations, such as the periplasm, leading to high protease activity. To avoid such circumstance, the level of protease expression and its activity must be tightly regulated. In case of VvsA, this intracellular check on its expression level might be controlled through the inhibitory role of VvsB. However, this same VvsB seemed to act differently in the extracellular milieu that leads to the production of active VvsA (Fig. 5).

Based on our findings, we propose that VvsA may undergo secretion into the extracellular environment in an intermediate state, retaining its C-terminal region. This intermediate form is then likely activated into its functional, active state with the assistance of VvsB. However, further research is necessary to thoroughly understand the precise mechanisms governing this process.

## Conclusions

**Figure 6.**
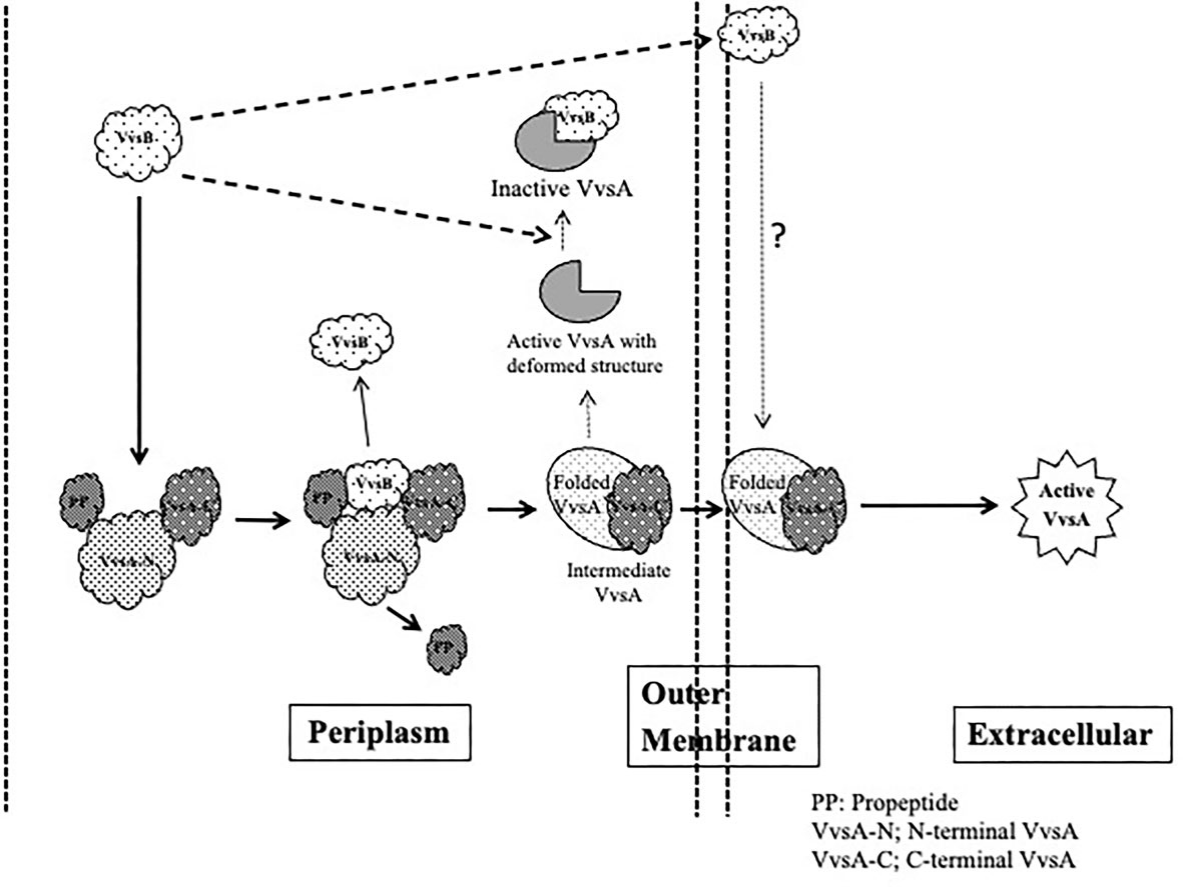
Schematic representation for the process of formation of active VvsA and action of VvsB in the process

Based on the results obtained, the role of VvsB in the activation of VvsA can be elucidated by the following hypothesis (Fig. 6): at first, VvsA transits through the outer membrane as an inactive intermediate of 59 kDa, then binds to the outer membrane surface and gradually converts into the active form of 45 kDa VvsA, as evidenced by its activity in the cellular membrane fraction which was explained by the activity in the cellular membrane fraction. At this stage, VvsB localized in the outer membrane might inhibit the conversion of VvsA intermediates to their active form (Fig. 3). Additionally, the active form of VvsA undergoes rapid inactivation through degradation (Kawase et al. 2022), but it didn't inhibit active VvsA and increased the serine protease activity (Fig. 5). Therefore, it was revealed that VvsB does not act as an inhibitor of VvsA activity in extracellular. However, given that VvsB is presumably an outer membrane protein, this experimental system was solely designed for the functional analysis of VvsA.The route shown by the solid line in Fig. 6 is obtained from the current results, while the dotted line represents the anticipated pathway.
